# Analysis of Breast Cancer Patients with T1-2 Tumors and 1-3 Positive Lymph Nodes Treated with or without Postmastectomy Radiation Therapy

**DOI:** 10.1038/s41598-020-66495-8

**Published:** 2020-06-18

**Authors:** Richard C. Gilmore, Mohamad E. Sebai, Kevin J. Psoter, Vishnu Prasath, Charalampos Siotos, Kristen P. Broderick, Lisa K. Jacobs, Susan C. Harvey, Mehran Habibi

**Affiliations:** 1 Department of Surgery, The Johns Hopkins University School of Medicine, Baltimore, USA; 2 Department of Pediatrics, The Johns Hopkins University School of Medicine, Baltimore, USA; 3 Department of Plastic and Reconstructive Surgery, The Johns Hopkins University School of Medicine, Baltimore, USA; 4 Department of Radiology and Radiological Science, The Johns Hopkins University School of Medicine, Baltimore, USA; 5 Division of Plastic & Reconstructive Surgery, Rush University Medical Center, Chicago, USA

**Keywords:** Breast cancer, Surgical oncology

## Abstract

The use of postmastectomy radiation therapy (PMRT) has been recommended for patients with 4 or more positive lymph nodes, however, its role in patients with 1-3 positive lymph nodes remains unclear. The purpose of this study is to evaluate oncological outcomes for breast cancer patients with T1-2 tumors and 1-3 positive lymph nodes after undergoing PMRT. We performed a single-institution retrospective investigation that evaluated the association between PMRT and outcomes in breast cancer patients with T1-2 tumors and 1-3 positive lymph nodes, who underwent mastectomy from 2004 to 2015. Multivariable Cox proportional hazards regression was used to evaluate the association of PMRT with disease-free survival and overall survival. A total of 379 patients met inclusion criteria, of which 204 (54%) received PMRT while 175 (46%) did not receive PMRT following mastectomy and were followed over a median of 5.2 years (25^th^–75^th^ percentile: 2.8–8.4 years). Recurrence was similar in patients receiving PMRT compared to those that did not: locoregional (0 vs 3, P = 0.061), distant (9 vs 3, P = 0.135) and any recurrence (11 vs 7, P = 0.525). After adjustment for potential confounding variables, PMRT was not associated with a statistically significant difference in disease-free survival (HR: 0.93; 95% CI: 0.48, 1.79) or overall survival (HR: 0.91; 95% CI: 0.45, 1.85). PMRT was not associated with improved oncological outcomes in patients with T1-2 breast cancer and 1-3 positive lymph nodes at our institution.

## Introduction

Consensus guidelines recommend the use of postmastectomy radiation therapy (PMRT) for patients with T3 and above tumors or T1-2 tumors with 4 or more positive lymph nodes and have advised against its use in patients with lymph node-negative disease^1,2^. However, the value of PMRT in patients with T1-2 tumors and 1-3 positive lymph nodes remains unclear.

A major contribution to this topic arose in 2014 when the Early Breast Cancer Trialists’ Collaborative Group (EBCTCG) published a meta-analysis on the effects of PMRT in this patient cohort^3^. This trial included 3786 women who underwent mastectomy and axillary nodal dissection, and had zero, one to three, or four or more positive lymph nodes identified. When PMRT was compared to mastectomy alone in the 1314 women with 1-3 positive lymph nodes, there was reduced locoregional recurrence (LRR), overall recurrence, and breast cancer mortality in patients who received PMRT. This study has been criticized for its high baseline rate of recurrence and its outcomes have been challenged in the current era of improved locoregional control and systemic therapy. We find this simply points out the importance of continued work in this area.

Similarly, other studies have found improved LRR and overall survival in patients with 1-3 positive lymph nodes who received PMRT^4–8^. McBride *et al*. found improved outcomes after PMRT in patients studied before the year 2000 but no difference in outcomes after that time, substantiating a claim of diminished effectiveness of radiation therapies in the era of modern adjuvant therapies^9^.

Due to the literature cited, our hypothesis was that, with modern systemic adjuvant therapies, the outcomes of patients who received PMRT and those who did not would be similar. The goal of our single-institution retrospective study was to evaluate whether PMRT was associated with improved oncological outcomes in patients with T1-2 tumors and 1-3 positive lymph nodes and to report outcomes in the group who received PMRT versus the group who did not, after controlling for confounding variables. Outcomes were defined as loco-regional (LRR), distant, and any recurrence, disease-free survival, and overall survival. If outcomes are unchanged, given the potential toxicity and costs associated with PMRT, there is the potential for a paradigm shift in care which could benefit patients by decreasing health-care costs and eliminating inessential therapies.

## Methods

We performed a retrospective investigation evaluating the association of receipt of PMRT with oncological outcomes in patients diagnosed with breast cancer with T1-2 tumors and 1-3 positive lymph nodes, who underwent mastectomy from 2004 to 2015. We excluded patients who had undergone neoadjuvant therapy of any form but patients with positive margins were not excluded. The study was approved by the Johns Hopkins School of Medicine institutional review board, which included a waiver for the obtainment of informed consent (IRB00046361). The methods and data management were Health Insurance Portability and Privacy Act (HIPPA) compliant.

### Inclusion criteria

Inclusion criteria for the study involved the following: females, over the age of 18, pathology-proven invasive breast cancer including ductal and lobular histology, primary surgical treatment with mastectomy, follow-up documentation available.

### End point ascertainment

The primary endpoints of interest were recurrence (locoregional, distant, and any recurrence), disease-free survival and overall survival.

### Treatment methods

Regarding PMRT, the selection of patients to be offered PMRT, radiation dose, use of bolus, and field arrangement was at the discretion of the treating radiation oncologist. Techniques for surgical mastectomy were stable over the interval from 2004 to 2015. Both skin sparing and nipple sparing surgical procedures were performed.

### Statistical analysis

Demographic and clinical characteristics between individuals who underwent mastectomy and received PMRT were compared to those that did not receive PMRT using Student t tests and Chi square or Fisher exact tests for continuous and categorical variables, respectively. Two-sample tests of proportions were used to compare recurrence outcomes. Kaplan Meier survival estimates were produced for disease-free survival and overall survival, by PMRT status, and compared using a log-rank test.

Univariate Cox proportional hazards regression was used to evaluate the association of PMRT and time to LRR, distant, and any recurrence, disease-free survival and overall survival. For these analyses, subjects entered risk sets upon initial date of diagnosis of breast cancer. Time to outcome was defined as time to death (overall survival), or disease free survival (defined as the minimum of time to death or any recurrence) as recorded in the Johns Hopkins tumor registry. Subjects not experiencing outcomes were censored at date of last clinical visit. Multivariable Cox proportional hazards was then used to evaluate the association of PMRT and time to each outcome after adjustment for the following covariates identified a *priori* based on previous literature: age at diagnosis, race (White vs. non White), tumor classification (T1 vs. T2), tumor grade ((1 and 2 vs. 3), receipt of hormonal therapy (yes/no), receipt of chemotherapy (yes/no) and estrogen and progesterone receptor status (negative vs. positive). Proportional hazards assumptions were tested for all models. Results of regression models are presented as hazard ratios (HR) with corresponding 95% confidence intervals (CI). A *P* value <0.05 was considered statistically significant. All analyses were performed using STATA Version 14.1 (StataCorp, College Station, TX).

### Compliance with Ethical Standards

Informed consent was waived by the institutional IRB for this study. The authors declare that they have no conflict of interest.

### Notes

The authors’ analyses comply with the laws in the United States of America. The datasets generated during and/or analyzed during the current study are not publicly available due to individual patient privacy laws which may be compromised but are available from the corresponding author on reasonable request.

## Results

A total of 379 patients met inclusion criteria, of which 204 (54%) received PMRT while 175 (46%) did not receive PMRT and were followed over a median of 5.2 years (IQR: 2.8–8.4 years).

**Figure 1:**
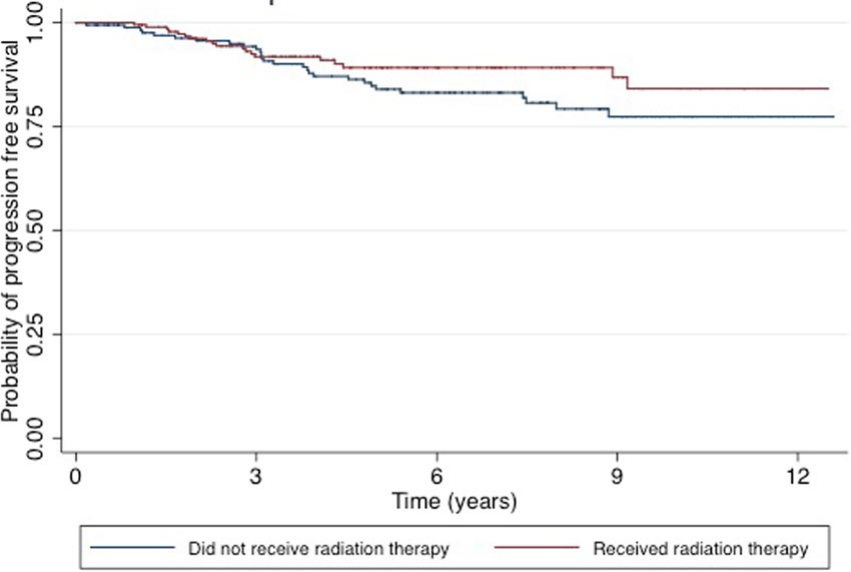
Kaplan-Meier overall survival estimates for our study population stratified by post-mastectomy radiation therapy receipt.

**Figure 2:**
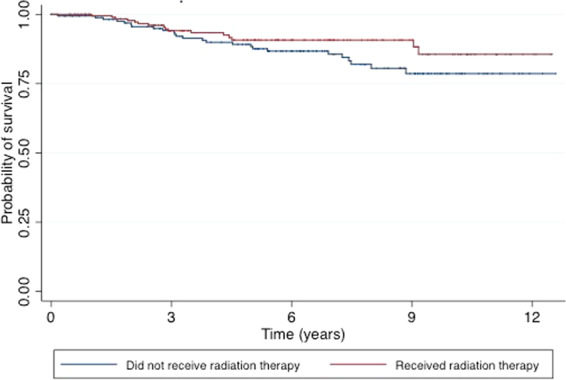
Kaplan-Meier disease-free survival estimate four our study population stratified by post-mastectomy radiation therapy receipt.

Table 1 compares the demographic and clinical characteristics of the study population, by PMRT status. Patients who received PMRT were younger (P = 0.043), and were more likely to receive adjuvant hormonal therapy (80.9 vs. 68.0%; P = 0.004) and chemotherapy (80.4 vs. 60.0%; P < 0.001). Outcomes in patients were similar by PMRT status; while a greater number of LRRs were observed in women who did not receive PMRT (3 vs 0, P = 0.061); fewer distant recurrences (3 vs. 9, P = 0.135) and fewer overall recurrences (7 vs 11, P = 0.525) were observed in women not receiving PMRT compared to those who did. Kaplan-Meier plots were produced for disease-free survival and overall survival (Figs. 1 & 2). After adjustment for potential confounding variables, receipt of PMRT was not associated with improved disease-free survival (HR: 0.93; 95% CI: 0.48, 1.79) or improved overall survival (HR: 0.91; 95% CI: 0.45, 1.85) (Table 2).

**Table 1:** Clinicopathologic characteristics of women who underwent mastectomy with T1-2 tumors and 1-3 positive lymph nodes from 2004–2015, by receipt of postmastectomy radiation therapy (PMRT).

	Did not receive PMRT (n = 175)		Received PMRT (n = 204)		*P* value
	*n*	%	*n*	%	
Age (years)					0.043
≤50 Years	71	40.6	104	51.0	
>50 Years	104	59.4	100	49.0	
Race					0.670
White	120	68.6	144	70.6	
Non-White	55	31.4	60	29.4	
Tumor classification					0.067
T1	92	52.6	88	43.1	
T2	83	47.4	116	56.9	
Grade					0.544
1 & 2	101	57.7	124	60.8	
3	74	42.3	80	39.2	
Hormonal therapy					0.004
No	56	32.0	39	19.1	
Yes	119	68.0	165	80.9	
Chemotherapy					<0.001
No	70	40.0	40	19.6	
Yes	105	60.0	164	80.4	
Estrogen receptor					0.394
Negative	30	17.1	42	20.6	
Positive	145	82.9	162	79.4	
Progesterone receptor					0.582
Negative	49	28.0	52	25.5	
Positive	126	72.0	152	74.5	
Lymphovascular invasion					<0.001
Not present	53	30.3	74	36.3	
Present	11	6.3	43	21.1	
Missing or unknown	111	63.4	87	42.7	
Scope of regional lymph node					0.153
None	3	1.7	0	0	
Biopsy or SLNB	30	17.1	51	25.0	
1-3 LNs removed	4	2.3	4	2.0	
4+ LNs removed	52	29.7	58	28.4	
SLNB and regular LN dissection	86	49.1	91	44.6	
Radiation treatment volume					<0.001
None	175	0	0	0	
Locoregional	0	0	102	50.0	
Comprehensive	0	0	102	50.0	

Patients with positive margins were not excluded.

**Table 2:** Multivariable Cox proportional hazards regression evaluating the association between PMRT and disease free and overall survival.

	Disease free survival		Overall survival	
	*Unadjusted* HR (95% CI)	*Adjusted*^*a*^ HR (95% CI)	*Unadjusted* HR (95% CI)	*Adjusted*^*a*^ HR (95% CI)
**Radiation therapy**				
No	REF	REF	REF	REF
Yes		0.93 (0.48, 1,79)		0.91 (0.45, 1,85)

^*a*^Adjusted for age at diagnosis, race (White vs. non White), tumor classification (T1 vs. T2), tumor grade ((1 and 2 vs. 3), receipt of hormonal therapy (yes/no), receipt of chemotherapy (yes/no) and estrogen and progesterone receptor status (negative vs. positive).

## Discussion

The efficacy of PMRT in T1-2 breast cancer patients with 1-3 positive lymph nodes remains unclear. In this study, we found no difference in the rates of recurrence, disease-free survival or overall-survival in breast cancer patients with T1-2 tumors and 1-3 positive lymph nodes who had been treated with or without PMRT.

Baseline LRR after mastectomy in patients who have not undergone PMRT has been shown to vary widely with rates between 4–20%^7,10–12^. At our institution, we encounter LRR in less than 2% of patients who underwent mastectomy and without PMRT. In addition, those who have compared LRR rates in PMRT vs no-PMRT groups have found variable results. Cosar *et al*. found 3% vs 17%, Huang *et al*. 3.1% vs 11%, McBride reported 3.4 vs 9.8 (early cohort) and 4.2 vs 2.8% (late cohort) and Tendulkar *et al*. 0% vs 8.9%^6,13–15^ (Table 3). Our study did not find a statistically significant difference in LRR between the two groups (3 vs 0, P = 0.061) with only 3 overall recurrences in our group of 379 women.

**Table 3:** LRR rates with or without PMRT in patients with 1-3 lymph nodes positive treated with adjuvant systemic therapy.

Authors	Accrual Dates	No. of Patients	Median Follow-Up (months)	Rate (PMRT vs. no PMRT, %)
Cosar *et al*.	1999–2006	90	72	3 vs. 17, p = 0.038
Huang *et al*.	1990–2008	318	102	3.1 vs. 11.0, p = 0.006
McBride *et al*.	1978–1997 2000–2007	505 522	205 84	3.4 vs. 9.5, p = 0.028 4.2 vs. 2.8, p = 0.48
Tendulkar *et al*.	2000–2007	369	62	0 vs. 8.9, p = 0.004

Data from the aforementioned EBCTCG trial suggests PMRT is effective in reducing LRR, overall recurrence and breast cancer mortality; however, recent evidence suggests these results may not be generalizable to patients in the current era of improved locoregional control and systemic adjuvant therapy, which contributes to a much lower baseline risk for recurrence. The 22 trials included in the EBCTCG were performed from 1964–1986. The 10-year LRR rate was 21.0% without vs 4.3% with PMRT (P < 0.001). Overall recurrence rates at 10 years were 45.5% without vs 33.8% with radiation (P < 0.001) and breast cancer mortality at 20 years was 49.4% without vs 41.5% with PMRT (P = 0.01, RR 0.78). In contrast, more recent studies with patient accrual beginning in the year 2000 have found loco-regional recurrence rates of less than 10%^4,9^. Results from the National Surgical Adjuvant Breast and Bowel Project (NSABP) found a LRR of 8.1% in patients with 1-3 positive lymph nodes with a median number of 16 lymph nodes dissected, compared with a LRR of 30% in the EBCTCG trial with a range of 7-11 lymph nodes dissected^3,16^. Our recurrence rate of <2% in patients not receiving PMRT is similar to recent studies such as that seen by McBride *et al*. in their late cohort^9^.

The explanation for lower recurrence rates in the current age is likely multifactorial^17^. Increased use and improved effectiveness of systemic treatment has been cited as one explanation for lower breast cancer recurrence rates in the modern era^18^. Chemotherapy regimens used prior to the turn of the century such as those utilized in the EBCTCG study were: cyclophosphamide plus methotrexate plus fluorouracil, methotrexate plus fluorouracil, or single-agent cyclophosphamide. Patients with hormone sensitive tumors were often given shortened (1-year) courses of tamoxifen and generally did not receive tamoxifen and chemotherapy in combination. Conversely, the modern era of systemic therapy for breast cancer includes the use of taxane-based chemotherapy regimens as well as the utilization of traztuzumab and pertuzumab for HER2 + tumors, longer (10-year) courses of tamoxifen for patients with hormone-sensitive tumors, as well as the use of aromatase inhibitors and combination therapy, all of which have likely contributed to lower recurrence rates in the current era.

In light of the controversy regarding the use of PMRT in this patient cohort, the American Society of Clinical Oncology, in combination with the American Society for Radiation Oncology and the Society of Surgical Oncology recently published a focused guideline update to address the risk/benefit ratio of PMRT in patients treated surgically with mastectomy having T1-2 tumors and 1-3 nodes positive at nodal dissection, based on the current literature^19^. They concluded that the available evidence as of 2016 shows PMRT reduces the risk of locoregional failure, recurrence, and breast cancer mortality in this patient population overall; however, they advised that certain subsets of patients are likely to have a sufficiently low risk of locoregional recurrence for which the risks of PMRT might outweigh the benefits; thus they encouraged the use of sound clinical judgment and decision-making on a case-by-case basis. Our study supports this approach and would suggest that more prospective randomized trials are necessary to determine the efficacy of PMRT in this patient population.

There are several limitations of our study. First, this is a retrospective study of 379 women where we are reporting the 12-year experience at our academic institution; therefore, results may be influenced by selection bias and may not be generalizable to all women who underwent mastectomy with T1-2 tumors and 1-3 positive lymph nodes during the same time period. For example, our reported rates of local recurrence are lower than those observed in previous investigations and our median 5-year follow up time may have limited capturing some recurrences. Similarly, temporal changes in adjuvant treatment such as the use of hormone therapy and chemotherapy occurred over the time period of our study and uptake of treatment at our Center may differ from others. A potential confounder could be the overall severity of the tumor because ‘worse’ tumors may be more aggressively treated with PMRT and chemotherapy due to elevated risk for distant metastases. Additionally, Her-2 receptor status was only collected beginning in 2010; the minimal number of cases during this time precluded meaningful analysis of this receptor’s status. Moreover, given incompleteness in our institutional tumor registry, lymphovascular invasion data was missing or unknown in a large proportion of our study population (n = 198, 52.2%), prohibiting us from meaningful analysis using this variable. Although there was also significant variability with regard to the extent of regional lymph nodes analyzed, this was not statistically significant (p = 0.153). On the other hand, there was significant variability with regard to radiation treatment volume with half of patients treated with PMRT receiving logoregional radiation (n = 102, 50.0%). and the other half receiving comprehensive radiation (chest wall + locoregional) therapy (n = 102, 50.0%). Standardization of radiation treatment volume would allow for more consistency in predictable treatment effect and would more precisely clarify generalizability of the result. In addition, due to the minimal number of recurrences observed (LRR, distant and any), we were unable to perform multivariable analyses of these variables where adjustment for patient level characteristics could be considered. Finally longer term follow-up is needed to sufficiently capture late recurrences.

## Conclusions

Our data suggest that PMRT is not associated with improved oncological outcomes in patients with T1-2 breast cancer and 1-3 positive lymph nodes. Further prospective randomized controlled trials are needed to analyze the effect of PMRT on outcomes.
